# Supplementary material for: Assessing the Effects of Landscape Change on the Occupancy Dynamics of the Greater White-Toothed Shrew Crocidura russula
Source: Life (Basel). 2022 Aug 14;12(8):1230. doi: 10.3390/life12081230 (PMC9409884; doi:10.3390/life12081230)
Supplement: Supplementary file 1 [file life-12-01230-s001.zip › life-1855169-supplementary.pdf]

Suppl. Table S1. Pearson correlations of 12 LiDAR variables (measured on 17 sampling plots) with the four first Principal Components (PCs) extracted from the PCA. The first PC (PC1) had significant correlations with nine out of 12 LiDAR variables, and the second (PC2) had four significant correlations.

VARIABLE	PC1	PC2	PC3	PC4
**CON 0,15-0,50**	-0,9615	-0,1764	-0,0642	0,0904
	p=,000	p=,498	p=,807	p=,730
**CON 0,50-1**	-0,8234	0,4652	0,1153	0,0163
	p=,000	p=,060	p=,659	p=,951
**CON 1-1,5**	-0,6162	0,7076	-0,2570	0,0572
	p=,008	p=,001	p=,319	p=,828
**CON 0,15-1,5**	-0,9852	0,0582	-0,0463	0,0774
	p=,000	p=,824	p=,860	p=,768
**CON 1,5-2,5**	-0,2355	0,8335	-0,4524	-0,1053
	p=,363	p=,000	p=,068	p=,688
**CON >2,5**	0,9739	-0,1569	0,0993	-0,0615
	p=,000	p=,548	p=,705	p=,815
**ALT 0,15-0,50**	-0,2150	0,7947	0,4900	0,1783
	p=,407	p=,000	p=,046	p=,493
**ALT 0,50-1**	0,7761	0,3302	-0,1715	0,0301
	p=,000	p=,196	p=,510	p=,909
**ALT 1-1,5**	0,3529	-0,3823	-0,7787	0,2307
	p=,165	p=,130	p=,000	p=,373
**ALT 0,15-1,5**	0,5931	0,7423	0,0071	0,1904
	p=,012	p=,001	p=,978	p=,464
**ALT 1,5-2,5**	0,7416	0,0315	0,1140	-0,5659
	p=,001	p=,905	p=,663	p=,018
**ALT >2,5**	0,7700	-0,0202	0,1806	0,4734
	p=,000	p=,939	p=,488	p=,055

LiDAR data technical parameters.

LiDAR sensor model	LEICA ALS 50
Flight start date	April-2016
Flight end date	August-2017
Minimum point density (p/m^2)	0.5
Average point density (p/m^2)	1-4.28
RMSE xy (m)	< 0.3
RMSE z (m)	< 0.2

The extraction of the LiDAR data was performed on circular plots with a radius of 53 m (8,834 m^2), obtained from the centre of each plot and equivalent to the square of 75 m x 75 m delimited by the four corners of the SEMICE sampling plots. For the two rectangular grids we calculated 125 m x 45 m areas.

LiDAR variables were grouped into two categories that provided information about vegetation vertical structure, and mean height.

The complexity of the vertical structure (6 variables: CON x-y) was estimated, as a percentage, by the relative contribution of each vegetation layer (0.15-0.50 m, 0.50-1 m, 1-1.50 m, 0.15-1.50 m, 1.50-2.50 m, > 2.50 m). The contribution of 0.15-1.50 m, 1.50-2.50 m and > 2.50 m layers sums 100. Contributions were calculated as the number of laser returns in each stratum divided by the total number of returns, excluding ground points and those at < 0.15 m. Thus, values close to 100 in the strata below 1.50 m indicate open canopies with lower layers comprising most of the total vertical structure, while values close to 100 in the strata above 1.50 m indicate dense tree crowns with limited presence of additional vertical structure at lower layers.

Vegetation mean height (6 variables: ALT x-y) was calculated as the mean of the heights of every laser return within each layer.
